# An Invited Reply to: A Comment on: The exploitation of sexual signals by predators: a meta-analysis (2022) White *et al.*

**DOI:** 10.1098/rspb.2023.0243

**Published:** 2023-05-10

**Authors:** Thomas E. White, Tanya Latty, Kate D. L. Umbers

**Affiliations:** ^1^ School of Life and Environmental Sciences, University of Sydney, Sydney 2106, Australia; ^2^ School of Science, Western Sydney University, Penrith, New South Wales 2751, Australia; ^3^ Hawkesbury Institute for the Environment, Western Sydney University, Penrith, New South Wales 2751, Australia

We are grateful for the thoughtful commentary of Bernal *et al*. [1], whose own contributions to questions of eavesdropping and signal evolution have been invaluable. In response to our recent meta-analysis on the subject [2] they raise several conceptual and methodological points, and while we agree—in part or in whole—on many, we do demur on others, which warrant a brief reply.

On a matter of both interpretation and method, Bernal *et al*. disagree with our findings that ‘*… contexts in which sexual signalling may incur no cost, or even reduce the incidence of predation, are common*’ ([1] quoting from [2]), and ‘*… in the wild there commonly is no risk from eavesdroppers*' ([1] attributed through paraphrase to [2]). To clarify, we did not make the latter claim, which is not consistent with the evidence we present. We instead argue for the opposite conclusion when we say, ‘Under more natural conditions we found a similarly moderate to strong average effect, suggesting a heightened risk to signallers in the wild.’ We do stand by the former claim, however, as a relatively uncontroversial statement of the observed variability of outcomes seen among the included studies. The conclusion is better understood in context, where the full phrase begins ‘*Variation in outcome measures was universally high, suggesting that …*’. This also highlights the possible underlying point of confusion: that of *average* effects versus *variation* in effects. These are distinct quantities, and we take care to differentiate between the conclusions implied by each [3]. To restate our central finding more directly in these terms, the current evidence suggests that, *on average*, the risk and cost of predatory eavesdropping is very high (solid confidence interval lines of figs 1 and 2 in [2]), but the considerable *variability* of reported outcomes shows that this is unlikely to be universally true; we should expect to find contexts—reported in future studies—with no eavesdropper-imposed costs in the wild (dashed prediction interval lines of figs 1 and 2 in [2]). If, however, we also understand Bernal *et al*. to be of the view that these findings are fundamentally at odds with ecological reality—and the costs of eavesdropping are universally positive and non-zero—then we suggest their argument is ultimately with the underlying cohort of experimental studies that we draw upon; a point that we discuss further below.

Continuing their concern for mis-specified average effects, Bernal *et al*. query our exclusion of effect size estimates in which either the control or treatment groups received zero attacks (but which we ultimately saw only among control groups), suggesting that this ‘… likely led to an underestimation of the predation risk on signalling individuals'. We remain confident that this analytical decision is well justified since, as explored in supplementary fig. 2 of [2] , standardized mean differences estimated from these assays stood in the range of *ca* 8–35. This would place them among the largest effects recorded [4] and are, we feel, biologically impossible. As discussed, the more parsimonious explanation is that the experimental controls in these studies—such as silent speakers stationed in open fields, which unsurprisingly attract no parasitoid attention [5]—are inappropriate for our question, and so warrant exclusion.

Bernal *et al*. [1] also discuss several methodological choices, such as our partitioning of studies into those that experimentally introduced graded or ‘continuous’ variation in signals between treatment and control groups, and those that used ‘discrete’ (presence/absence) manipulations. This was chiefly an attempt to explore gross differences in methodology as a source of between-study heterogeneity, which we were glad to see Bernal *et al.* [1] agree was warranted. There is further confusion as to how this was achieved, however, as Bernal *et al*. [1] characterize our classification of studies as ‘*… when organisms are presented with a single stimulus (discrete manipulation) …*’ and ‘*… eavesdroppers presented with two forms of a signal (continuum [sic] manipulation) …*’. This is incorrect and is not how we scored studies nor described doing so, since the order of stimulus presentation (i.e. singly or simultaneously) had no bearing on our classification. In eavesdropper-preference assays, both stimuli were presented to predators and were available simultaneously in forced-choice contexts, irrespective of whether the experimenters used a ‘discrete’ or ‘continuous’ manipulation of signals. In eavesdropping-risk assays, the stimuli were also simultaneously available, albeit in a field setting, but this was again independent of the nature of the manipulation. With respect to the ecological interpretation of our moderator analysis, then, we do feel that key differences between these two forms of manipulation include stimulus conspicuousness and identity—as discussed—though we certainly agree with Bernal *et al.* that predator classification, learning and cognition more generally would ultimately all bear on any identified differences between studies (which we did not find).

Bernal *et al*. [1] add valuable nuance to the question of modality-specific risk, though suggest that ‘*A main claim of the meta-analysis is based on the assumption that eavesdropping risk is modality-specific …*’. This was not an assumption we made, however, but rather a longstanding prediction we sought to test [6,7]. Our findings ultimately affirmed it—contingent on the available evidence—though we have no argument with Bernal *et al.*'s discussion of the complexities of modality-specific differences in signalling ecology (but see below), nor the long-understood reality that the physical properties of signals are not the only determinant of their active space [8].

We also agree that predator and prey behaviour are important mediators of eavesdropping interactions in the wild. This is a point to which we could dedicate only a paragraph, while cautioning that our compiled estimates ‘*… can be understood as representing the baseline risk of predation absent any adaptations for actively enhancing and/or subverting the privacy of communication*’. For a deeper discussion, we too refer readers to Bernal *et al*.'s [9] excellent recent review on the topic.

Finally, and more broadly, Bernal *et al*.'s [1] repeated emphasis on the context-dependence and complexity of eavesdropping interactions—arising from differences in behaviour, cognition, taxonomy and/or signalling ecology—and their note of caution that the findings of meta-analyses are ‘*… only as good as its inputs …*’ are entirely fair, though they echo well-trodden critiques of quantitative syntheses ([10, pp. 413–422]). The implied overarching concern, as also discussed in the medical literature they cite, is that the underlying systems and studies are so unique, variable, and/or rich in nuance as to be difficult to compare in the context of meta-analysis, thereby rendering the core results of such syntheses highly tentative, if not misleading. Our points here are twofold and draw on extensive discussions in the evidence synthesis literature to which we refer interested readers [3,10,11].

One is that we fully acknowledge the existence of ecologically important differences between included studies and the systems they describe. This is true of almost every synthesis in evolutionary ecology [12], in part because direct replications remain vanishingly rare in our field [13]. The accepted remedy to this anticipated variability is to explicitly acknowledge and examine it, as we do, in part by using random effects models to account for non-independence arising from study-level and phylogenetic differences, reporting heterogeneity (*I*^2^) and prediction intervals, directly testing hypothesized sources of such heterogeneity, and interpreting high among-study variability as a result unto itself, which is often of equal or greater interest and importance than mean effects [14]. The contextual differences Bernal *et al*. detail will—among other sources—have contributed to our high estimates of among-study variability, which we examined within the limited scope of a single work. As is common [12], the source of much heterogeneity remains to be explained, which presents exciting fodder for future empirical studies and/or syntheses [11].

The second related but more fundamental point is that if the outcomes of individual studies, and the dynamics of the signalling systems they describe, are so rich in context-specific subtlety as to be incomparable in the context of such a synthesis, then this is true irrespective of whether it is a formal qualitative or quantitative synthesis, a narrative review, or the intuitive assessment of our state of understanding we each hold as individual researchers. That is, it precludes inductive inference more broadly, and so we can claim little understanding of the generalities of signal exploitation and eavesdropping based on the results of disparate studies of aphid sex pheromones, guppy displays, cricket song or frog choruses, as has been attempted to great effect in the past [6,15,16]. Despite their differences and simplifying assumptions, we hold that the studies included in our synthesis are using comparable designs to answer the same overarching question—are sexual signals vulnerable to exploitation by predators?—and note that this is explicitly affirmed in most of the studies themselves.

This judgement, however, among myriad others, is ultimately for meta-analysts to justify and readers to evaluate. This highlights a central value-proposition of systematic approaches to synthesis in behavioural and evolutionary ecology. The transparent reporting and justification of search methods and analytical decisions, inclusion criteria and assessment of underlying study quality, and open archival of data and code, afford readers the opportunity to appraise a given work and the conclusions that it draws regarding our current understanding. As Bernal *et al*. [1] demonstrate, this can encourage fruitful discussions and highlight future research foci, which will hopefully contribute to the resolution of exciting eco-evolutionary questions.

## Data Availability

This article has no additional data.
